# Ketone Bodies Promote Amyloid-β_1–40_ Clearance in a Human in Vitro Blood–Brain Barrier Model

**DOI:** 10.3390/ijms21030934

**Published:** 2020-01-31

**Authors:** Romain Versele, Mariangela Corsi, Andrea Fuso, Emmanuel Sevin, Rita Businaro, Fabien Gosselet, Laurence Fenart, Pietra Candela

**Affiliations:** 1 Laboratoire de la Barrière Hémato-Encéphalique (LBHE), UR 2465, Université d'Artois, F-62300 Lens, France; romain_versele@ens.univ-artois.fr (R.V.); mariangela_corsi@libero.it (M.C.); emmanuel.sevin@univ-artois.fr (E.S.); fabien.gosselet@univ-artois.fr (F.G.); laurence.tilloy@univ-artois.fr (L.F.); 2 Department of Medico-Surgical Sciences and Biotechnologies, Sapienza University of Rome, Corso della Repubblica 79, 04100 Latina, Italy; rita.businaro@uniroma1.it; 3 Department of Experimental Medicine, Sapienza University of Rome, Dip. di Chirurgia “P. Valdoni”, Via A. Scarpa 16, 00161 Rome, Italy; Andrea.Fuso@uniroma1.it

**Keywords:** blood–brain barrier, Alzheimer’s disease, ketone bodies, β-hydroxybutyrate, acetoacetate, amyloid-β peptide

## Abstract

Alzheimer’s disease (AD) is characterized by the abnormal accumulation of amyloid-β (Aβ) peptides in the brain. The pathological process has not yet been clarified, although dysfunctional transport of Aβ across the blood–brain barrier (BBB) appears to be integral to disease development. At present, no effective therapeutic treatment against AD exists, and the adoption of a ketogenic diet (KD) or ketone body (KB) supplements have been investigated as potential new therapeutic approaches. Despite experimental evidence supporting the hypothesis that KBs reduce the Aβ load in the AD brain, little information is available about the effect of KBs on BBB and their effect on Aβ transport. Therefore, we used a human in vitro BBB model, brain-like endothelial cells (BLECs), to investigate the effect of KBs on the BBB and on Aβ transport. Our results show that KBs do not modify BBB integrity and do not cause toxicity to BLECs. Furthermore, the presence of KBs in the culture media was combined with higher MCT1 and GLUT1 protein levels in BLECs. In addition, KBs significantly enhanced the protein levels of LRP1, P-gp, and PICALM, described to be involved in Aβ clearance. Finally, the combined use of KBs promotes Aβ efflux across the BBB. Inhibition experiments demonstrated the involvement of LRP1 and P-gp in the efflux. This work provides evidence that KBs promote Aβ clearance from the brain to blood in addition to exciting perspectives for studying the use of KBs in therapeutic approaches.

## 1. Introduction

Affecting more than 46 million individuals worldwide, Alzheimer’s disease (AD) is now considered to be the most common cause of dementia in the elderly population (World Health Organization (WHO)), with an increase of 7.7 million new cases every year. The pathological features of AD include intracellular neurofibrillary tangles (reviewed in [1]) and the accumulation of amyloid-β peptides (Aβ) forming senile plaques in brain parenchyma as well as in the walls of intracerebral microvessels [2]. Aβ peptides are ~4 kDa proteins comprising 36–48 aa. Around brain microvessels, the most abundant form of Aβ comprises 40 aa (Aβ_1–40_ ~80–90%) [3]. Among several independent mechanisms for clearing these Aβ peptides from the central nervous system (CNS), the bidirectional transport of Aβ across the blood–brain barrier (BBB) plays a major role [4,5,6].

The BBB is a physiological barrier histologically formed by endothelial cells (ECs) lining the brain microvessels [7,8]. This physical barrier strictly controls the exchange of molecules between the brain and the blood in order to protect the CNS and to maintain its functioning and homeostasis [8,9]. ECs express specific sets of receptors and transporters at the apical side (blood side) and basolateral side (brain side), regulating the brain’s nutrient supply. Glucose transporter 1 (GLUT1) and monocarboxylate transporter 1 (MCT1) are respectively involved in importing glucose and ketone bodies (KBs) into the brain. Furthermore, ECs express several receptors and transporters involved in the bidirectional soluble Aβ exchange across the BBB. Low-density lipoprotein receptor-related protein 1 (LRP1) is involved in Aβ efflux (brain to blood) [10,11], while the receptor for advanced glycation endproducts (RAGE) is implicated in Aβ influx (blood to brain) [12,13]. Moreover, several members of the ATP-binding cassette (ABC) family, such as AΒCB1 (also known as P-glycoprotein (P-gp)) and AΒCG2 (also known as breast cancer resistance protein (BCRP)), are involved in Aβ efflux and limiting Aβ influx [13,14]. Dysregulation of their expression may trigger the progressive cerebral and vascular accumulation of Aβ, as demonstrated in animal studies and clinical research [5,6,15]. Notably, the downregulation of LRP1 and P-gp during AD is one of the key events leading to Aβ accumulation by decreasing Aβ clearance from the brain [11,16]. These two proteins have been functionally linked to phosphatidylinositol-binding clathrin assembly protein (PICALM) [17], which is identified as a genetic risk factor for AD [18,19]. Therefore, increasing the clearance of Aβ across the BBB via the induction of P-gp or/and LRP1 expression, and consequently PICALM, may be an effective strategy to protect the brain from the accumulation of Aβ [20] and prevent AD onset. One of the new AD treatments currently under development is the ketogenic diet (KD) [21,22,23,24]. KD is characterized by a nutrient intake that is very poor in carbohydrates and high in fatty acids [25]; hence, it produces much of the same physiological effects of low food intake (i.e., fasting) [26,27] and promotes the production of KBs (in liver mitochondria), β-hydroxybutyrate (βHB) and acetoacetate (AcAc) in particular. During starvation or consumption of a KD, the relative level of glucose in the plasma declines, and that of KBs increases [25,27]. The KBs are then utilized by extrahepatic tissues—including the brain—as an energy source [28,29]. Indeed, KBs are transported across the BBB via MCT1 and consumed by the brain as the major energy source when glucose is limited [30,31]. Despite experimental and clinical evidence supporting the protective effect of a KD against AD [32,33] through reduced Aβ deposition and improved learning memory [34,35,36,37], little is known about the impact of this diet on BBB function.

Notably, the way in which a KD counteracts amyloid deposition remains unstudied. Therefore, we hypothesized that a KD and, in particular, KBs, act at the BBB level to promote amyloid elimination from the brain. Hence, we investigated the effects of relevant KBs using a human in vitro BBB model. Our data show that KBs do not modify the BBB integrity but impact the expression of proteins involved in the transport of KBs and glucose (MCT1 and GLUT1, respectively) as well as Aβ_1–40_ peptide transport across the BBB. In particular, KBs enhanced protein expression of LRP1, P-gp, and PICALM at the BBB level. The combined use of KBs (AcAc and βHB) improved basolateral-to-apical Aβ_1–40_ peptide transport through the BBB. Inhibition studies also demonstrated the functional involvement of LRP1 and P-gp in the clearance of Aβ.

## 2. Results

### 2.1. KBs do not Affect the Viability and Integrity of BLECs

**Figure 1:**
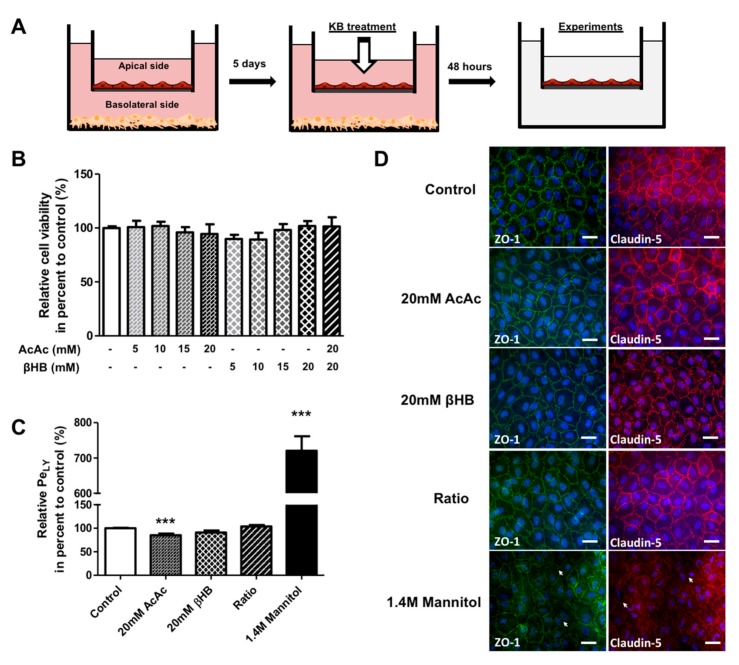
The effect of KBs on BLEC permeability. (**A**) Schematic representation of the human in vitro BBB model used for the KB study. This model is composed of an apical compartment in the filter containing endothelial cells and a basolateral compartment in the well with brain pericytes. After 5 days of co-culture, BLECs were incubated with various concentrations of KBs, AcAc, or βHB alone (5–20 mM) or in in combination (20 mM AcAc/20 mM βHB) for 48 h. Following KB treatment, the BLECs on the insert were transferred to a new plate for experiments. (**B**) The effects of KBs on cell viability were analyzed by MTT assay. (**C**) BLEC monolayer integrity was determined by measuring the endothelial lucifer yellow permeability (Pe_LY_). Each bar represents the mean ± SEM relative to the control conditions (Pe_LY_ = 0.92 ± 0.03 × 10^−3^ cm∙min^−1^). The results are representative of three independent experiments performed in triplicate (*** *p* < 0.001). (**D**) Associated tight junction protein ZO-1 (green) and tight junction protein claudin-5 (red) staining were stained using immunofluorescence. Interruptions in the staining are indicated by white arrows. Nuclei were stained with Hoechst reagent and appear in blue. Scale bar: 50 µm.

To determine the effects of KBs on the BBB, we used a human in vitro BBB model consisting of co-cultivated endothelial cells derived from umbilical cord blood CD34^+^ stem cells with brain pericytes [38,39]. These cells were seeded on a matrigel-coated insert that separates two compartments, the apical compartment with endothelial cells (blood side) and the basolateral compartment with the pericytes (brain side) (Figure 1A). After 5 days of culture, the endothelial cells showed BBB properties similar to those observed in vivo, and were named brain-like endothelial cells (BLECs).

BLEC viability was determined using an MTT assay after 48 h of exposure to different KB concentrations in the apical compartment (corresponding to the blood side). The results shown in Figure 1B reveal no difference in viability between KB-treated cells and control (non-treated) cells. Based on these results, 20 mM AcAc, 20 mM βHB, and 20 mM AcAc/20 mM βHB (referred to as the “ratio” in the rest of the paper) were selected as the treatment conditions for further experiments. Since the BBB integrity is of primary importance for maintaining correct brain functioning, we assessed the impact of KBs on BBB permeability. To this end, BLECs were incubated with KBs for 48 h. To exclude the possibility that BLECs were not responsive to the damage possibly induced by KBs, we treated the human in vitro BBB model with mannitol, which is known to disrupt the BBB [40]. BBB permeability was checked by measuring the speed of diffusion of the small paracellular marker Lucifer Yellow (LY; ~400 Da) across the BLEC monolayer to determine the endothelial permeability of Lucifer Yellow (Pe_LY_). As shown in Figure 1C, no significant differences in Pe_LY_ values were observed for any of the KBs tested compared to the control, except for the 20 mM of AcAc treatment where a decrease in Pe_LY_ was observed (14.6%; *p*-value ≤ 0.0001). As expected, a huge and significant increase in Pe_LY_ values for the mannitol treatment compared to non-treated cells (620.6%; p-value ≤ 0.0001) was observed. In addition, immunofluorescence analysis of tight junction proteins ZO-1 and claudin-5 confirmed that KBs did not affect the intercellular contacts between endothelial cells (Figure 1D). By contrast, mannitol treatment caused an interruption in the staining for ZO-1 and claudin-5 protein, indicating an alteration in the tight junction network (Figure 1D, basal lane).

Overall, our results indicate that KBs do not cause toxicity to BLECs and do not modify BBB integrity, at least in the tested concentration range.

### 2.2. KBs Increase MCT1 and GLUT1 Protein Levels in BLECs

**Figure 2:**
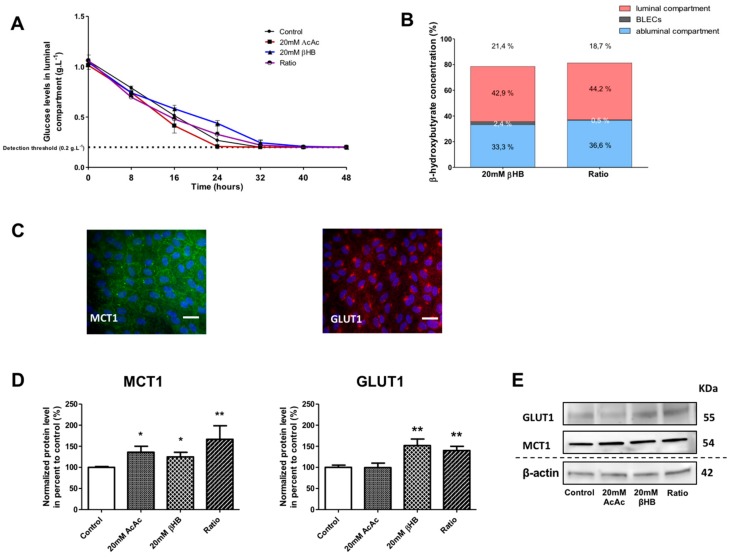
Effect of KBs on the human in vitro BBB model. (**A**) BLECs were incubated with various concentrations of KBs for 48 h: 20 mM AcAc, 20 mM βHB, or ratio (20 mM AcAc/20 mM βHB). The glucose concentration was measured in the apical compartment every 8 h. (**B**) The βHB concentration was measured in the apical and basolateral compartments and in BLECs. The data are represented as mean ± SEM obtained from three independent experiments. (**C**) The presence of MCT1 (green) and GLUT1 (red) in the BLECs was performed using immunostaining. Nuclei were stained with Hoechst reagent, and appear in blue. Scale bar: 50 µm. (**D**) The effects of KBs on MCT1 and GLUT1 protein levels were analyzed by Western blot. The protein level was normalized using β-actin. The protein data represent the mean ± SEM obtained from at least three independent experiments relative to control conditions (* *p* < 0.05, ** *p* < 0.01). (**E**) The images are representative of at least three independent experiments.

During starvation, when glucose levels decline and blood KB concentrations increase, an increase in GLUT1 and MCT1 expression is observed in neuronal tissues [41,42]. Therefore, we wanted to determine whether MCT1 and GLUT1 at the level of BLECs would be modulated by KB treatment. First, glucose and βHB concentrations were analyzed in the culture media of our in vitro BBB model in the presence of different KBs amounts over 48 h, with untreated cells used for comparison. The concentration of AcAc was not measured as we lacked a method for AcAc detection. As shown in Figure 2A, a decrease in glucose concentration, from 1 to 0.2 g∙L^−1^ (detection threshold of Precision Xtra meter), was observed in the apical compartment. Similar results were observed in the basolateral compartment (data not shown). βHB concentrations were similarly measured in (i) the apical compartment, (ii) BLECs, and (iii) the basolateral compartment after 48 h (Figure 2B).

The stability of the βHB at 37 °C and at 5% CO_2_ was checked in the culture media over 48 h (see the methods section). After incubation with 20 mM βHB in the BBB model, we observed 42.9% of the total amount of βHB in the apical compartment, 2.4% in BLECs, and 33.3% in the basolateral compartment. Hence, 21.4% of the initially added βHB was not detectable. The results with 20 mM AcAc/20 mM βHB (ratio condition) were similar to those using 20 mM βHB (Figure 2B). The latter results demonstrate that under experimental conditions in which the glucose levels fall, KBs were partially catabolized by BLECs and were still present in the culture medium 48 h after treatment. Under the same conditions, we examined the effects of KBs on the MCT1 and GLUT1 protein levels in BLECs. First, immunofluorescence staining demonstrated that both MCT1 and GLUT1 were expressed in untreated BLECs (Figure 2C). Next, quantification of MCT1 and GLUT1 was performed using Western blot assays. After 48 h of KB treatments, MCT1 protein levels significantly increased in AcAc, βHB, and the ratio condition by 35.9% (*p*-value = 0.0247), 24.9% (*p*-value = 0.0455), and 66.5% (*p*-value = 0.0062), respectively, in the BLECs as compared to the control (Figure 2D). In the same way, GLUT1 protein levels increased significantly in βHB and in the ratio condition by 51.9% (*p*-value = 0.0027) and 39.8% (*p*-value = 0.0047), respectively.

Overall, our results demonstrate that when glucose levels fall, βHB is stable and is still present in the culture medium and inside the BLECs after 48 h of treatment. In addition, the presence of KBs in the culture medium was associated with higher MCT1 and GLUT1 protein levels in BLECs, which corresponds with previous in vivo results.

### 2.3. KBs Modify the Levels of Proteins Involved in Aβ_1–40_ Peptide Transport Across the BBB

**Figure 3:**
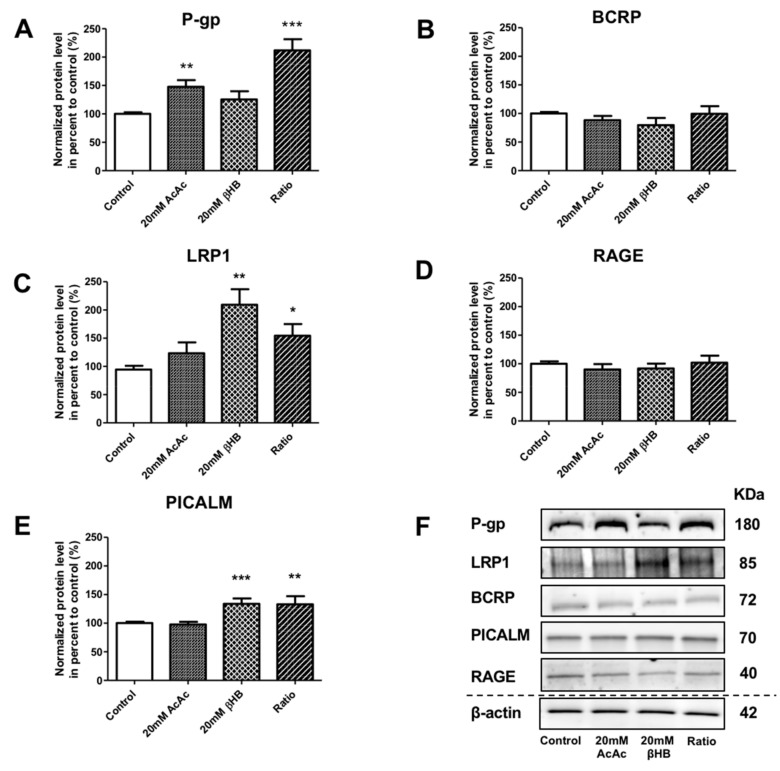
Effects of KBs on the protein expression of actors involved in Aβ peptide transport in BLECs. The protein levels of (**A**) P-gp, (**B**) BCRP, (**C**) LRP1, (**D**) RAGE, and (**E**) PICALM were quantified using Western blot. The data were normalized using β-actin. The results represent the mean ± SEM obtained from at least three independent experiments relative to the control conditions (* *p* < 0.05, ** *p* < 0.01, *** *p* < 0.001). (**F**) The images are representative of at least three independent experiments.

In addition to the effect of KBs on MCT1 and GLUT1, we assessed the protein levels of receptors and transporters involved in the apical-to-basolateral and basolateral-to-apical transport of Aβ_1–40_ peptide in BLECs following KB treatment. We showed that the protein levels of P-gp were significantly increased in AcAc and in the ratio treatments by 47.9% (*p*-value = 0.0024) and 112.0% (*p*-value ≤ 0.0001), respectively, compared to the control (Figure 3A). BCRP protein levels were not significantly different (Figure 3B). Interestingly, LRP1 protein levels were significantly upregulated in the presence of βHB and in the ratio treatments by 109.3% (*p*-value = 0.0079) and 54.5% (*p*-value = 0.0317), respectively, compared to the control (Figure 3C). RAGE protein levels were not affected by any of the KBs treatments (Figure 3D). We also investigated whether KBs affected the levels of PICALM protein, which has been described to interact with LRP1 and P-gp transporters [17,43]. As observed in Figure 3E, PICALM protein levels increased in BLECs upon exposure to βHB and ratio by 39.1% (*p*-value = 0.0009) and 39.4% (*p*-value = 0.0031), respectively, compared to the control.

These data indicate that KBs are able to modulate LRP-1, P-gp, and PICALM protein levels in BLECs. These three proteins are the major players involved in Aβ peptide efflux across the BBB.

### 2.4. KBs Increase Basolateral-to-Apical Aβ Peptide Transport Through the BBB with the Involvement of LRP-1 and P-gp

**Figure 4:**
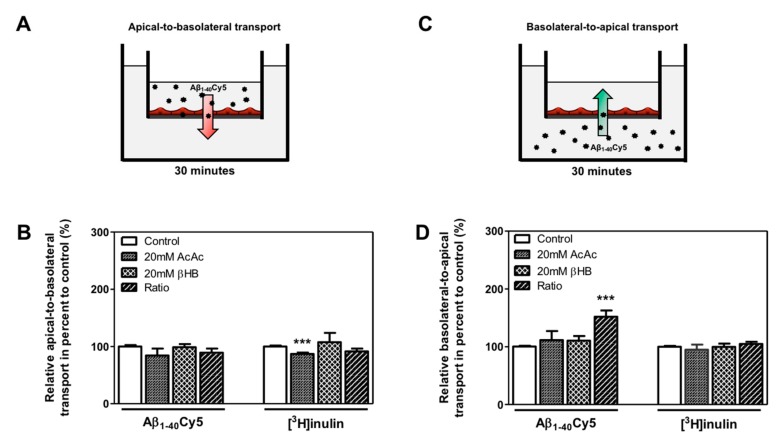
Effect of KBs on Aβ transport across BBB. (**A**) Schematic representation of the human in vitro BBB model used for (**A**) apical-to-basolateral (red arrow) and (**C**) basolateral-to-apical (green arrow) Aβ transport. After 48 h of treatment with KBs, Aβ_1–40_Cy5 or [^3^H]inulin was added to the (**B**) apical compartment or (**D**) basolateral compartment, incubated for 30 min, and permeability was then assessed. The data represent the mean ± SEM obtained from at least three independent experiments relative to the control conditions, each of which was performed in triplicate (*** *p* < 0.001).

We hypothesized that KB treatment could be associated with a higher clearance of Aβ peptide through the BBB. Thus, the apical-to-basolateral (influx) and basolateral-to-apical (efflux) Aβ_1–40_Cy5 peptide transport across BLECs was assessed as previously described [13,39,44]. For these experiments, we used Aβ_1–40_, since this is the most abundant amyloid peptide found in brain microvessels [45]. As a control in these experiments, the apical-to-basolateral and basolateral-to-apical transport of inulin was determined across BLECs. Inulin was used as a non-specific transport marker, as previously described [13,46]. As shown in Figure 4B, the apical-to-basolateral transport of the Aβ_1–40_Cy5 peptide was not affected by the presence of KBs. In the same way, the values for [^3^H]inulin transport did not differ significantly in the presence of KBs, except for the AcAc condition, where a slight decrease was observed (12.9%, *p*-value = 0.0005) (Figure 4B).

**Figure 5:**
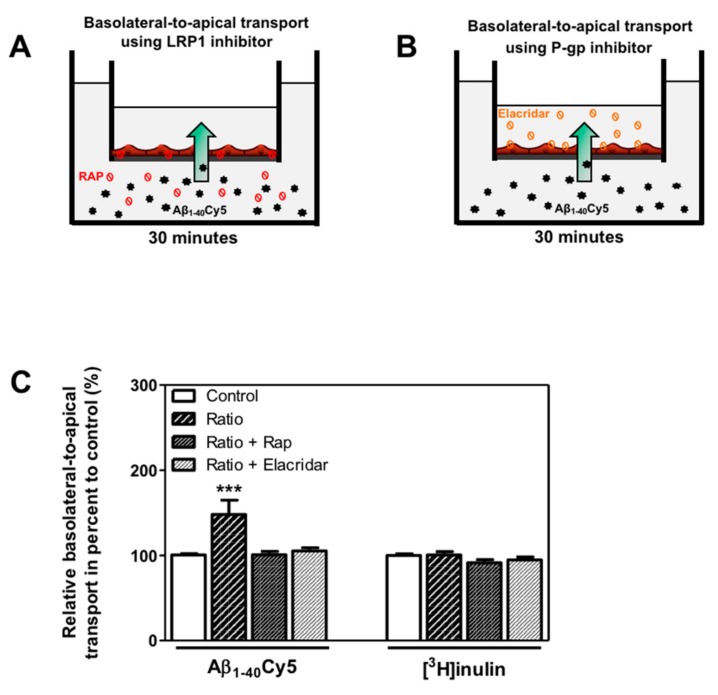
Involvement of LRP1 and P-gp in basolateral-to-apical transport of Aβ_1–40_Cy5 peptide after ratio KB treatment. (**A**) Schematic representation of the human in vitro BBB model used for basolateral to-apical Aβ transport (green arrow) in the presence of (**A**) LRP1 or (**B**) P-gp inhibitors, respectively RAP (red ban symbol) or elacridar (orange ban symbol). (**C**) Aβ_1–40_Cy5 or [^3^H]inulin basolateral-to-apical transport was performed with RAP or elacridar. The data represent the mean ± SEM obtained from at least three independent experiments relative to the control conditions, each of which was performed in triplicate (*** *p* < 0.001).

Only the basolateral-to-apical transport of Aβ_1–40_Cy5 (Figure 4D) was significantly higher in the ratio treatment relative to the control condition (by 52.0%, *p*-value ≤ 0.0001), suggesting that KBs are able to partly enhance basolateral-to-apical Aβ peptide transport. In fact, no significant difference in Aβ_1–40_Cy5 transport was observed in the other KB conditions. The values for [^3^H]inulin transport did not differ significantly in the presence of KBs (Figure 4D), confirming that Aβ transport across the BBB is regulated by specific mechanisms. In light of our previous results (Figure 3A,C) after 48 h of KB treatments (ratio), the transport of Aβ_1–40_Cy5 or [^3^H]inulin was studied in the presence or in the absence of two standard inhibitors for LRP1 and P-gp [11,47,48,49], the receptor-associated protein (RAP) (in the basolateral side) or elacridar (in the apical side), respectively. As shown in Figure 5C, the increased basolateral-to-apical of Aβ_1–40_Cy5 peptide transport was abolished in the presence of RAP or elacridar. By contrast, the basolateral-to-apical transport of [^3^H]inulin was not affected by RAP or elacridar. The treatments had no significant effect on BBB permeability (data not shown), confirming that the BBB model was not compromised by KBs.

These observations suggest that only the combination of the two KBs was able to increase basolateral-to-apical transport of Aβ peptide. The use of LRP1 and P-gp inhibitors demonstrated the functional involvement of LRP1 and P-gp activity in the efflux of Aβ peptide across the BBB.

## 3. Discussion

For a few years now, there has been an increased focus on the role of the transport of Aβ peptides across the blood–brain barrier (BBB) [4,50,51]. It appears that increasing the clearance or reducing the entry of Aβ peptides in the brain could be a promising therapeutic approach for counteracting the pathogenic processes observed in AD [52]. In recent years, there has been mounting interest in the possible utilization of a ketogenic diet (KD) or ketone body (KB) supplementations in the treatment of AD [53,54]. On the basis of KBs’ beneficial effects on the CNS, and the lack of published data on the BBB, we addressed the question of whether KBs could modulate the expression of receptors/transporters implicated in the transport of Aβ across the BBB and whether these changes could impact functional transport. To explore our hypothesis, we used a human in vitro BBB model composed of a co-culture of endothelial cells (derived from CD34^+^ cells) and brain pericytes [38]. This model has already been used to study the transport of several molecules, including Aβ [39], and represents a useful tool for deciphering the cellular and molecular mechanisms of this transport. Firstly, we examined the effect of KBs on cell viability and BBB integrity. No cell toxicity was observed in our model following 48 h of treatment with a range of AcAc or βHB concentrations (0–20 mM alone, or 20 mM AcAc/20 mM βHB). This is consistent with previous studies that showed 95% of cell viability when PC12 cells were exposed in 0–20 mM βHB [55], and in contrast with other studies that reported an increase in BBB integrity when mouse brain microvascular endothelial cells were exposed to increasing concentrations of either βHB or AcAc [56]. Our results further show that KBs do not modify BBB permeability and continuous tight junction networks at cellular borders. Interestingly, a decrease in Pe_LY_ was observed under treatment with AcAc. It is likely that AcAc could change some structural component of the BBB, and in doing so, reinforce the permeability of the barrier [57]. This possibility needs to be clearly addressed through further investigations. In the absence of cellular toxicity, we decided to conduct mechanistic studies using highest doses. The used concentrations are higher than would be expected in vivo [25,58], but also shorter in duration than those used in vivo [59]. In addition, the choice is even more pertinent knowing that an alternative to the KD is the use of exogenous KBs and ketone sources [60]. These exogenous KBs induce a nutritional ketotic state similar to that derived from KD, resulting in elevated serum KBs, but are safer and more efficient [61,62]. Moreover, higher doses are possible [63]. Therefore, it is of great interest to carry out in vitro experiments with KBs at higher concentrations to assess their molecular effects on cells.

We showed that when the glucose concentration decreases, it fell rapidly from 1g∙L^−1^ (the normal approximate blood sugar levels found in vivo) to zero, while βHB was stable and still present in the culture medium and in endothelial cells (we found that 42.9% of the total amount of βHB was in the apical compartment (~8.5 mM), 2.4% was in the BLEC (~0.5 mM), and 33.3% (~6.6 mM) was in the basolateral compartment). The presence of KBs both in the culture medium and in BLECs after 48 h of incubation corresponded with the higher levels of MCT1 and GLUT1 proteins observed in BLECs. These observations are in agreement with those published by others in which an increase of KBs in the blood following a KD was associated with higher MCT1 and GLUT1 expression in rat brain microvessels [64,65,66,67], and in contrast with other studies, which reported that a KD does not affect the expression of GLUT1 at the BBB level [68]. In addition, it has been shown that βHB is specifically able to enhance GLUT1 protein expression in mouse brain endothelial cells [69].

Previous reports have shown that the induction of ketosis in mouse and dog models led to reduced accumulation of Aβ within the brain [37,70]. However, the way in which KBs are able to decrease the Aβ burden has yet to be elucidated. For the first time, we demonstrated that KBs are able to modulate LRP-1, P-gp, and PICALM protein levels in BLECs. In particular, we showed that (i) the protein levels of P-gp were significantly increased in AcAc and in the ratio condition, (ii) LRP1 protein levels were significantly upregulated by both βHB and in the ratio condition, and. interestingly, that (iii) the PICALM protein level was induced in BLECs upon exposure to βHB and the ratio condition. Instead, the protein levels of RAGE and BCRP were not affected by the addition of KBs. These data are in line with previous studies demonstrating that nutritional factors (e.g., a cholesterol-enriched diet or oleocanthal (a phenolic secoiridoid component of extra virgin olive oil and vitamin B) exert neuroprotective effects against AD by impacting the transcription of genes coding for receptors and transporters involved in Aβ transport across the BBB [71,72,73,74].

In particular, most of the cited papers showed that the upregulation of LRP1 and/or P-gp is essential for promoting Aβ clearance across the BBB [75]. According to these results, we also demonstrated the marked elevation of basolateral-to-apical Aβ_1–40_ Cy5 transport in the presence of KBs in the ratio condition. In addition, using our BBB model, we confirmed that these proteins are implicated in the efflux of Aβ peptide. Our experiments demonstrate that the presence of RAP (in the basolateral compartment) and elacridar (in the apical compartment) in the BBB model was associated with significantly lower basolateral-to-apical transport of Aβ. These observations agree with similar in vitro results obtained in mouse models and human endothelial cells showing that upregulation of LRP1 and P-gp promotes Aβ clearance across the BBB [11,67,74,76]. It is now accepted that the concerted Aβ clearance of LRP1 and P-gp is linked by PICALM. Indeed, using a transgenic mouse model, it was demonstrated that the reduction of PICALM expression aggravates Aβ pathology [77]. Inversely, the presence of PICALM in brain endothelial cells is essential for the rapid LRP1/P-gp-mediated clearance across the BBB [17]. In line with these results, we also observed that KBs are able to enhance PICALM levels. Thus, enhanced LRP1 and P-gp efflux function by KBs could also be mediated, at least in part, by activating PICALM expression. The significance of inducing this pathway was previously reported in another study showing that PICALM deficiency in mice diminished Aβ efflux across the BBB, whereas PICALM re-expression was necessary to reverse this mechanism [77]. According to the fundamental role of PICALM in the transcytosis of Aβ, it would be interesting to know whether KBs are able to regulate trafficking inside the cells. Future studies will address this question.

However, as already mentioned, it cannot be excluded that other receptors or transporters are involved in the clearance of Aβ peptide [10]. For example, it has been shown that other ABC transporters, such as BCRP, have an important role in Aβ clearance from the brain [13,14,78,79]. However, no changes were detected in the expression of BCRP after KB treatment. For this reason, the implication of BCRP in the basolateral-to-apical Aβ_1–40_Cy5 transport was excluded.

On the other hand, no change in the apical-to-basolateral Aβ_1–40_Cy5 transport was observed under our culture conditions, confirming that KBs have no modulating effect on RAGE protein expression. These data partially agree with those of Guo et al. showing that the active form of vitamin D (1,25-dihydroxyvitamin D3) has an important role in increasing Aβ transport from the brain to the blood via the upregulation of LRP1 and the downregulation of RAGE [80]. Indeed, while the implication of RAGE to mediate the entry of circulating Aβ is undisputed [12,13,76], it is possible that not all exogenous molecules may be able to modulate the protein expression of this cellular receptor. In this sense, the ability of rifampicin and caffeine to induce LRP1 and P-gp expression in wild-type C57BL/6 mice was recently demonstrated, while the treatments had no effect on RAGE expression in microvessels [75].

Interestingly, only a combination of KBs markedly increased basolateral-to-apical Aβ_1–40_Cy5 transport. This indicates that the combined effects of the two KBs are more efficient or different than the effect elicited by each KB. Previous in vitro studies have already shown a different mechanism of action of AcAc and βHB. For example, using mouse brain microvascular-derived endothelial cells, it was demonstrated that AcAc and βHB differentially regulate the permeability of the BBB [56]. AcAc but not βHB stimulates the production of the vasoconstrictor endothelin-1. By contrast, βHB—but not AcAc—increases the synthesis of vascular endothelial growth factor (VEGF), a potent modulator of vascular permeability [56]. Moreover, Cheng et al. reported that AcAc and βHB have opposite effects on human endothelial cell viability, with AcAc inhibiting and βHB promoting cell proliferation [81]. More recently, Kanikarla-Marie and Jain reported that unlike βHB, high concentrations of AcAc intensify endothelial cell oxidative stress [82], inducing TNFα and MCP-1 expression as well as ROS accumulation [83]. Therefore, in accordance with our in vitro results, it is quite possible that the effects of the two KBs are synergistic and different than the effects of the KBs in isolation, highlighting the importance of studying both their separate and combined effects on cells. Increasing evidence indicates that alterations in DNA profiles can lead to changes in gene expression [84,85]. Knowing that KBs and, in particular, βHB are specific inhibitors of class I histone deacetylases (HDACs), it is likely that KBs provoke HDAC inhibition, which increases histone acetylation and thereby induces transcriptomic and epigenetic modifications at the BBB level, including the transport of Alzheimer’s-associated peptides, such as amyloid. Future studies are needed to determine whether KBs can affect epigenetic and molecular events in the brain and in particular, at the BBB level.

In conclusion, this work provides evidence that KBs are not toxic on BLECs and can promote Aβ_1–40_ transendothelial transport (efflux) via their ability to enhance the function of LRP1 and P-gp transporter (and PICALM), which are responsible for Aβ clearance. These results may explain the recovery of cognitive functions observed in animal models as well as in patients. Moreover, the increased Aβ_1–40_ transendothelial transport detected in BLECs in the presence of 20 mM βHB/20 mM AcAc suggests that the KBs’ combined effect is greater than the individual effects. Our work provides exciting perspectives for studying the KBs as a non-pharmacological means of targeting Aβ clearance and developing new therapeutic strategies in modulating the progression of AD.

## 4. Materials and Methods

### 4.1. Materials

Endothelial cell medium (ECM; Sciencell, USA) supplemented with 5% heat-inactivated fetal calf serum (FCS, GIBCO, Life Technology SAS, Saint Aubin, France), 50 µg·mL^−1^ gentamicin (Biochrom GmbH, Germany), and 0.5% endothelial cell growth factor (Sciencell, USA) (ECM-5) was prepared and stored at 4 °C for a maximum of one week. Beta-hydroxybutyric acid (βHB, as DL-β-hydroxybutyric acid sodium salt) and acetoacetate (AcAc, as lithium acetoacetate) were purchased from Sigma-Aldrich (Lyon, France), dissolved in culture medium (according to the manufacturer’s instructions), and stored at –20 °C. Lucifer yellow (LY, Lucifer Yellow CH dilithium salt) and human serum albumin (HSA) were purchased from Sigma-Aldrich. [^3^H]inulin (1.25 Ci. mmol^−1^) was purchased from Analytical Sciences (Waltham, MA, USA). Fluorescent human amyloid beta-peptide (1–40)-Cy5 labeled (Aβ_1-40_Cy5) was purchased from Phoenix Pharmaceuticals (Strasbourg, France). The powder was resuspended in 250 µL of DMSO (Sigma-Aldrich, France) followed by an addition of 750 µL of RH-HSA 0.1% to obtain a solution at 1 µM, according to the manufacturer’s instructions, and stored at –20 °C to be used at 10 nM in RH-HSA 0.1% buffer. Receptor-associated protein (RAP Human, Recombinant, E.coli; 553506) was purchased from Merck Millipore, dissolved in RH-HSA 0.1%, and freshly used. Elacridar was purchased from Sigma-Aldrich (United Kingdom), dissolved in DMSO at 0.5 mM, and stored at -20 °C to be used at 0.5 µM in RH-HSA 0.1% buffer.

### 4.2. The Human in Vitro BBB Model

The human brain-like endothelial cells (BLECs) from the in vitro BBB model were from the co-culture of endothelial cells (ECs) derived from CD34^+^ cord blood hematopoietic stem cells with brain pericytes as previously described by Cecchelli et al. (2014) [38]. Donors’ parents, in accordance with French legislation, gave their consent for the collection of human umbilical cord blood. The French Ministry of Higher Education and Research (CODECOH DC2011-1321) approved the collection of human cells. According to the method described by Pedroso et al. (2011) [86], CD34^+^ stem cells were isolated from human umbilical cord blood and then prompted to differentiate into ECs. For the differentiation step, isolated CD34^+^ were cultured in endothelial cell growth medium-2 (EGM-2; Lonza) supplemented with 20% (v/v) FCS (Life Technologies) and 50 ng·mL^−1^ of vascular endothelial growth factor (VEGF) (PrepoTech Inc), on 0.2% gelatin-coated 24-well plates (2 × 10^5^ cells/well). After 15–20 days of differentiation, ECs were amplified in the culture dish at P2 and frozen in frozen medium (10% DMSO, 50% FCS, ECM-5). Then, ECs were thawed and seeded onto Matrigel (BD Biosciences, San Jose, CA, USA)-coated filters (Costar Transwell inserts, Corning Inc., Corning, NY, USA, pore size 0.4 µm, 8 × 10^4^ cells/insert), and co-cultured with bovine brain pericytes isolated as described by Vandenhaute et al. (2011) [87] in a 12-well plate with ECM-5. The culture medium was changed every two days. These culture conditions were maintained for 5 days and enabled the CD34^+^-ECs to acquire a BBB phenotype (i.e., BLECs). Under these conditions, the model was ready for experiments and stable for 21 days. A mycoplasma detection (Lonza, Rockland, ME, USA) was performed to validate the absence of mycoplasma in the cell culture medium.

The co-culture model delimits two compartments, the apical compartment that mimics the blood side and the basolateral compartment that mimics the brain side. The BBB model was described in Cecchelli et al. (2014) [38].

### 4.3. Treatment with KBs

After 5 days of co-culture, AcAc and/or βHB were added to the apical compartment (blood side) for 48 h at 37 °C. The concentrations used (5, 10, 15, and 20 mM AcAc or βHB or both in ratio 20 mM AcAc/20 mM βHB) were selected according to the KB levels measured during a normal diet (basal serum level < 0.3 mM), a ketogenic diet or starvation (5–8 mM), and diabetic ketoacidosis (> 25 mM) [58,88]. Since blood AcAc and βHB are normally present in the blood during fasting or starvation at a 1:1 ratio, we also treated cells with 20 mM AcAc/20 mM βHB [89]. The pH of the cell culture medium with or without KBs was measured during experimental conditions (48 h at 37 °C and 5% CO_2_) using a pH meter (WTW inolab pH level 1). In these conditions, the pH of the cell culture media was monitored before (~7.3) and after (~6.9) KB treatments. No significant pH variation was observed after KB treatment compared to the control.

### 4.4. Cell Viability Assay

Cell viability was determined using the standard MTT [3-(4,5-dimethylthiazol-2-yl)-2,5-diphenyltetrazolium bromide] assay (AR1156, Boster Biological Technology, Pleasanton, CA, USA). For each condition tested, cells were seeded in triplicate and incubated during 48 h in the absence or in the presence of different concentrations of KBs (5, 10, 15, 20 mM of each, or in ratio 20 mM AcAc/20 mM βHB) once the BBB phenotype was established. Following treatments, 150 000 BLECs/insert were counted for each condition. MTT was added to the apical compartment for 2 h at 37 °C and 5% CO_2_, and the purple formazan crystals from MTT were then dissolved with DMSO (300 µL/well). The optical density (OD) was measured at a wavelength of 570 and 630 nm using a microplate reader (Synergy H1, BioTek. Colmar, France). The % relative cell viability compared to the control condition was calculated using the following formula: % relative cell viability = ((OD_570_ - OD_630_) condition)/((OD_570_–OD_630_) control) × 100.

### 4.5. Measurement of glucose and βHB levels in culture medium

After 5 days of co-culture, 20 mM AcAc or 20 mM βHB or 20 mM AcAc/20mM βHB (ratio treatment) was added to the apical compartment for 48 h at 37 °C and 5% CO_2_. During this time, the glucose and βHB concentrations were measured in both the apical compartment and the basolateral compartment. The Precision-Xtra meter (Abbott Labs, Abbott Park, IL, USA) with a glucose or β-ketone sticks was used. In these assays, the glucose and βHB concentrations measured by the sticks were representative of the glucose and βHB present in the culture medium. Data were expressed in mmol·L^−1^ for βHB and in g·L^−1^ for glucose. The βHB concentration within BLECs was measured at 48 h following the treatment. Briefly, after medium removal, BLECs were washed twice with RH buffer and lysed with RIPA lysis buffer (Millipore Corporation, Burlington, MA, USA). The βHB concentration in the BLECs was normalized by the protein amount using Bradford assay (Bio-rad, Munich, Germany). The amount of βHB into the supernatant was assayed using β-ketone sticks, as described above. The βHB concentration was verified in the culture medium without cells at 37 °C and 5% CO_2_ to ensure the stability of βHB.

### 4.6. Evaluation of BLEC Monolayer Permeability

The integrity of the BLEC monolayer after 48 h of treatment with KBs was evaluated using the method described by Dehouck et al. (1992) [90]. Each coculture condition was performed in triplicate. The fluorescent integrity marker LY was used at a final concentration of 50 µM in RH buffer to obtain the endothelial permeability coefficient (Pe) from the apical-to-basolateral compartment. The fluorescence of LY in each compartment was measured using the excitation/emission wavelength (432/538 nm) using a microplate reader (Synergy H1). In this calculation method, both the filter without cell permeability (PSf = insert filter + matrigel coating) and filter plus cell permeability (PSt = insert filter + matrigel coating + ECs) were taken into account, according to the formula: 1/PSe = 1/PSt + 1/PSf, where PS is the permeability x surface area product (in microliters per minute) obtained by dividing the volume cleared from the donor to the receiver compartment (in µL) for 1 h. Themass balance (%) was calculated from the amount of compound recovered in the donor and receiver compartment at the end of the experiment divided by the total amount added in the donor compartment at time zero. To be taken into account for Pe determination, the mass balance should be between 80% and 120%.

### 4.7. Immunostaining

After 48 h of treatments with or without KBs, BLECs were fixed and permeabilized as described in Table 1. Then, a 30-min blocking with PBS-CMF (0.2 g·L^−1^ KH_2_PO_4 ,_ 8.0 g·L^−1^ NaCl, 2.87 g·L^−1^ Na_2_HPO_4_-12H_2_O, and 0.2 g·L^−1^ KCl) with 10% (v/v) normal goat serum (NGS) was performed. Then, cells were incubated for 60 min with the primary antibody in PBS-CMF supplemented with 2% NGS at room temperature (RT), against the target protein as described in Table 1.

After three washes in PBS-CMF supplemented with 2% NGS, the cells were incubated with secondary polyclonal antibody (goat anti-rabbit Alexa 568, A11036, Molecular Probes, Eugene, OR, USA) diluted 1:500 in PBS-CMF supplemented with 2% NGS in the dark for 30 min at RT. Hoechst 33358 was used for nuclei staining. After mounting with Mowiol (Sigma-Aldrich) containing 1,4-diazabicyclo [2.2.2]octane (Sigma-Aldrich), images were taken using a Leica microscope (DMRD; Leica Microsystems, Wetzlar, Germany) and processed using ImageJ32 software.

### 4.8. Protein Extraction and Immunoblots

After 48 h of treatment, BLECs were washed twice with cold RH buffer and scraped with 50 µL UT4 lysis buffer (7M Urea, 2M Thiourea, 4% CHAPS) or 50µL RIPA 1X lysis buffer (10 mM Tris-Cl (pH 8.0), 1 mM EDTA, 0.5 mM EGTA, 1% Triton X-100, 0.1% sodium deoxycholate, 0.1% SDS, 140 mM NaCl) (Merck Millipore, USA) supplemented with a cocktail of protease and phosphatase inhibitors purchased from Sigma-Aldrich (1% protease inhibitors cocktail P8340; 1% phosphatase inhibitors cocktail 1 P2850; 1% phosphatase inhibitors cocktail 2 P5726). Lysates were then centrifuged at 10,000 rpm for 10 min at 4 °C. The supernatant was saved and sonicated three times at 40 W for 5 s. The lysate protein concentration was measured using the Bradford method. Then, 30 µg of total protein for LRP1 or 20 µg for the other proteins were mixed with Laemmli (Bio-rad, Munich, Germany) supplemented with beta-mercaptoethanol (Sigma-Aldrich, Germany) and boiled for 5 min at 95 °C. Details of the denaturation and reduction step of the protein extract are described in Table 2.

Then, the protein mixes were electrophoresed using Criterion™ TGX™ (Tris-Glycine eXtended) precast gels (Bio-rad) and subsequently electrotransferred to nitro cellulose membranes (GE Healthcare, Germany). Nonspecific binding sites were blocked by incubating the membrane with Tris-buffered saline 0.1% Tween 20 (TBST; 20 mM Tris–HCl, pH 8.0, 500 mM NaCl, 0.1%Tween 20) supplemented with 5% skimmed milk at RT for 1 h under agitation. Membranes were incubated in the appropriate primary antibodies (Table 2) and incubated at 4 °C overnight, excepted for P-gp (2 h at RT) and β-actin (20 min at RT). Then, membranes were washed with TBST three times for 5 min each, and then incubated with the horseradish peroxidase conjugated secondary antibody anti-mouse (1:3000, Dako/Agilent Technologies, Inc., Santa Clara, CA, USA,) or secondary antibody anti-rabbit (1:8000, Dako/Agilent Technologies, Inc., Santa Clara, CA, USA) for 1 h at RT. Then, membranes were washed with TBST three times for 5 min each and bands of immunoreactive protein were visualized after membrane incubation with enhanced chemiluminescence (GE Healthcare) reagent and revealed by the Western blot Imaging system Azure c600 (Azure Biosystems, Dublin, Ireland). Quantification of the relative densities of bands was performed with TotalLab TL 100 1D Gel Analysis software (Nonlinear Dynamics, Newcastle, UK). The protein expression normalization was conducted with anti-β-actin. Studies were performed at least three independents experiments, with two Western blots per experiment.

### 4.9. Amyloid-β (_1-40_) Peptide Transport Studies

Forty-eight hours after KB treatments, the apical-to-basolateral and the basolateral-to-apical transport of Aβ_1-40_ across BLECs was investigated as previously described [13,44,46] (Briefly, for apical-to-basolateral studies, filters were transferred to new 12-well plates containing 1.5 mL of RH-HSA 0.1% (the receiver solution) per well. In the insert corresponding to the apical compartment, 0.5 mL of RH-HSA 0.1% supplemented with 10 nM Aβ_1-40_Cy5 peptide was added (the donor solution). For basolateral-to-apical studies, filters were transferred to 12-well plates containing 1.5 mL of RH-HSA 0.1% supplemented with 10 nM fluorescent Aβ_1-40_Cy5 (the donor solution). In the apical compartment, 0.5 mL of RH-HSA 0.1% (the receiver solution) was added. In parallel, the apical-to-basolateral and the basolateral-to-apical transport of 400 nM [^3^H]inulin was performed in the same way as the Aβ_1-40_Cy5 peptide transport, thus representing a paracellular marker. The BBB integrity was checked by measuring the Pe_LY_ as described in Section 4.6. For LPR1 inhibition studies, the basolateral-to-apical transport of Aβ_1-40_ transport was performed by adding 10 nM of Aβ_1-40_Cy5 peptide with or without 200 nM of RAP in the basolateral compartment. For P-gp inhibition studies, the basolateral-to-apical transport of Aβ_1-40_ transport was performed by adding 10 nM of Aβ_1-40_Cy5 in the basolateral compartment with or without 0.5 µM of elacridar in the apical compartment. All transport studies were performed in triplicate on a rocking platform at 37 °C for 30 min. At the end of the incubation period, aliquots of the donor and receiver solutions were collected, and the fluorescence compounds (Aβ_1-40_Cy5 and LY) were measured with a spectrofluorimeter (Synergy H1). The radioactivity of [^3^H]inulin was measured using a scintillation counter (Hidex 300 SL, Hidex, Turku, Finland). The permeability (apparent permeability coefficient, cm.sec^−1^) of Aβ_1-40_-Cy5 or [^3^H]-inulin was calculated according to the following equation: (1) Papp = (k.V(R))/(A.60) as previously described [91]. k was the transport rate (min^−1^) defined as the slope obtained by linear regression of the cumulative fraction absorbed (FAcum) as a function of time (min), V(R) was the volume in the receiver chamber (mL), and A was the area of the filter (cm^2^). Determination of the cumulative fraction absorbed (amount permeated), FAcum versus time, FAcum was calculated from: (2) FAcum = ∑ (C(Ri))/(C(Di)), where C(Ri) was the receiver concentration at the end of the interval i and C(Di) was the donor concentration at the beginning of interval i. A linear relationship should be obtained. The apical-to-basolateral and the basolateral-to-apical Papp were determined by Equation (1) for each molecule. For each filter, the calculated mass balance was determined as described in Section 4.6. Experiments were performed at least 3 times.

### 4.10. Statistical Analysis

All statistical analyses were performed using the GraphPad Prism 5.01 statistical software package (GraphPad Software, Inc., San Diego, CA, USA). Data are presented as the mean ± standard error of the mean (SEM). The results of experiments were evaluated using the Mann Whitney t-test. The threshold for statistical significant was set to * *p* < 0.05, ** *p* < 0.01, or *** *p* < 0.001.

## Figures and Tables

**Table 1:** Antibodies used for the immunostaining experiment.

Protein Target	Antibody Reference	Fixation/Permeabilization	Antibody Dilution
Claudin-5	34-1600 (Invitrogen)	Ice-methanol 30’’	1:100
GLUT1	07-1401 (Merck Millipore)	Ice-methanol 30’’	1:100
MCT1	AB3538P (Merck Millipore)	Ice-methanol 30’’	1:100
ZO1	61-7300 (Invitrogen)	Paraformaldehyde 1% 10’/Triton X100 0,1% 10’	1:200

**Table 2:** Antibodies used for the Western blot experiment.

Protein Target	Antibody Reference	Lysis Buffer	Special Condition	Antibody Dilution	Size (KDa)
β-actin	A5541 (Sigma Aldrich)	RIPA	-	1:20000	42
BCRP	Ab3380 (Abcam)	RIPA	Without heat denaturation	1:1000	72
GLUT1	07-1401 (Merck millipore)	RIPA	-	1:1000	55
LRP1	5A6 (Santa Cruz)	UT4	Without reduction / β-mercaptoethanol	1:200	85
MCT1	Ab179832 (Abcam)	RIPA	-	1:1000	54
P-gp	C219 (Gene Tex)	RIPA	Without heat denaturation	1:500	180
PICALM	HPA019053 (Sigma Aldrich)	RIPA	-	1:1250	70
RAGE	Ab37647 (Abcam)	RIPA	-	1:1000	40

## Abbreviations

Aa	Amino acid
AcAc	Acetoacetate
AD	Alzheimer’s disease
Aβ	Amyloid β
BBB	Blood-brain barrier
BCRP	Breast cancer resistance protein
βHB	β-hydroxybutyrate
BLECs	Brain-like endothelial cells
CNS	Central nervous system
DMSO	Dimethyl sulfoxide
ECM	Endothelial cell medium
EGM-2	Endothelial cell growth medium-2
FCS	Foetal calf serum
GLUT1	Glucose transporter 1
HSA	Human serum albumin
KBs	Ketone bodies
KD	Ketogenic diet
LRP1	Low-density lipoprotein receptor-related protein 1
LY	Lucifer yellow
MCT1	Monocarboxylate transporter 1
NGS	Normal goat serum
Pe	Endothelial permeability
PBS-CMF	Phosphate-buffered saline calcium– and magnesium–free
P-gp	P-glycoprotein
RAGE	Receptor for advanced glycation endproducts
RAP	Receptor–associated protein
RH	Ringer-HEPES
RT	Room temperature
SEM	Standard error of the mean
TBST	Tris-buffered saline 0.1% Tween 20
ZO–1	Zonula occludens–1
